# Serum Total Bilirubin and Risk of Cancer: A Swedish Cohort Study and Meta-Analysis

**DOI:** 10.3390/cancers13215540

**Published:** 2021-11-04

**Authors:** Maria J. Monroy-Iglesias, Charlotte Moss, Kerri Beckmann, Niklas Hammar, Goran Walldius, Cecilia Bosco, Mieke Van Hemelrijck, Aida Santaolalla

**Affiliations:** 1 Translational Oncology & Urology Research (TOUR), King’s College London, London SE1 9RT, UK; maria.j.monroy_iglesias@kcl.ac.uk (M.J.M.-I.); charlotte.moss@kcl.ac.uk (C.M.); kerri.beckman@unisa.edu.au (K.B.); Cecilia.bosco@kcl.ac.uk (C.B.); mieke.vanhemelrijck@kcl.ac.uk (M.V.H.); 2 Cancer Research Institute, University of South Australia, Adelaide, SE 5001, Australia; 3 Epidemiology Unit, Institute of Environmental Medicine, Karolinska Institute, 171 77 Stockholm, Sweden; niklas.hammar@ki.se (N.H.); goran.walldius@ki.se (G.W.)

**Keywords:** cancer risk, hyperbilirubinemia, bilirubin, cohort study, lung cancer, colorectal cancer, breast cancer, gynecological cancer, melanoma

## Abstract

**Simple Summary:**

Several studies published to date have shown inconclusive results in the association between serum bilirubin and risk of site-specific cancer types and overall cancer. Therefore, there is a need to further investigate this association. Data from the large Swedish Apolipoprotein Mortality Risk (AMORIS) cohort study was used. We found that overall high levels of bilirubin had no association with overall cancer risk. However, a positive association was found between melanoma and breast cancer risk. On the other hand, an inverse association was found between high levels of bilirubin and risk of gynecological and lung cancers. Further studies are required to establish if bilirubin can be used as a biomarker for risk assessment and/or as a novel therapeutic target.

**Abstract:**

Bilirubin has strong antioxidant properties that have been hypothesized to be preventive against the development of cancer. Thus, we aimed to investigate the association between serum total bilirubin (STB) and risk of overall and site-specific cancers in the large Swedish Apolipoprotein Mortality Risk (AMORIS) cohort. We also performed a systematic review and meta-analysis for specific cancer types (colorectal, breast and lung). We found no association between high levels of STB and risk of overall cancer. Regarding site-specific cancer, there was an inverse association between increased STB and lung cancer (Hazard Ratio (HR) for the 4th quartile (Q4) vs. Q1: 0.50; 95%CI: 0.44–0.59) and gynecological cancer (HR for Q4 vs. Q1: 0.86; 95%CI: 0.76–0.99). A positive association was found with melanoma (HR for Q4 vs. Q1: 1.25; 95%CI: 1.06–1.47) and breast cancer (HR for Q4 vs. Q1: 1.13; 95%CI: 1.01–1.25) risk. The meta-analysis showed an inverse association between high levels of STB and risk of lung cancer (Relative risk (RR): 0.69; 95%CI: 0.55–0.86). No associations were seen for colorectal and breast cancer risk. Further studies are required to establish if bilirubin can be used as a biomarker for risk assessment and/or as a novel therapeutic target.

## 1. Introduction

**Figure 1:**
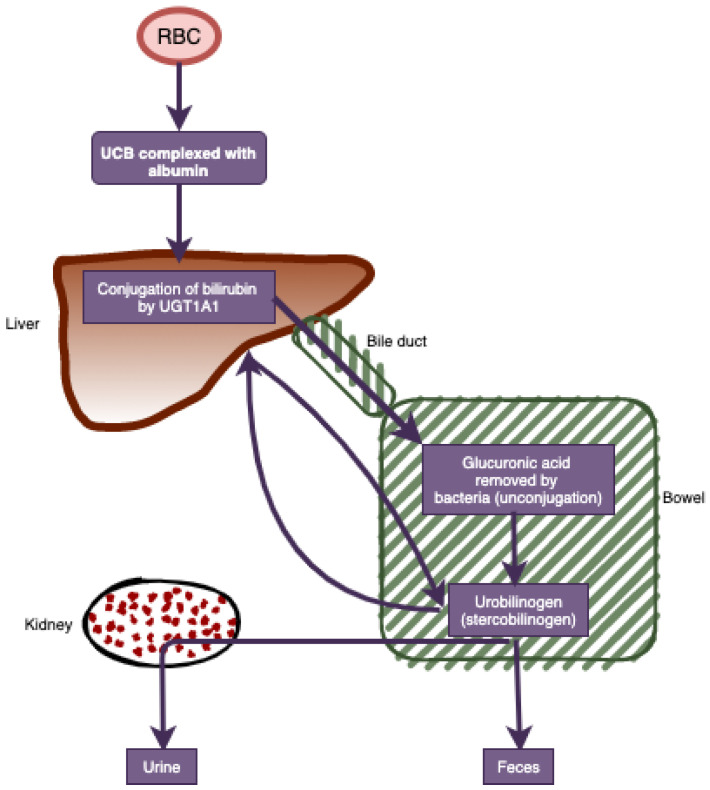
Heme degradation pathway. RBC, red blood cells; UCB, unconjugates bilirubin; UGT1A1, diphosphoglucuronyltransferase.

It has been proposed that endogenous bile pigments, such as bilirubin, may play a protective role against the development of a variety of chronic diseases, including cancer, due to their potent anti-inflammatory and antioxidant properties [1]. Serum total bilirubin (STB) is derived primarily from the degradation of hemoglobin and transported to the liver by binding to albumin [1]. Blood levels of total bilirubin consist of primarily unconjugated bilirubin (UCB). In vitro, UCB has been found to be the most active antioxidant component of bilirubin [2,3]. After the liver removes UCB from the blood, it is conjugated by a uridine diphosphoglucuronyltransferase (UGT1A1) and transported to the bowel via the bile. Here, it is unconjugated by bacteria and either excreted or reabsorbed [4] (Figure 1). Conjugation (or glucuronidation) by UGT1A1 is essential for the elimination of bilirubin, and thus, a polymorphism in the UGT1A gene results in the accumulation of UCB in the serum (hyperbilirubinemia) [5]. This mild unconjugated hyperbilirubinemia is known as Gilbert’s Syndrome and has a prevalence of 5–10% in Caucasians [5].

STB is commonly used as a biomarker of hepatobiliary and hematopoietic diseases [6]. Of late, bilirubin has been discussed in the setting of disease prevention due to growing evidence of its potent anti-inflammatory and antioxidant properties [7,8,9]. Given that oxidative stress and inflammation are involved in the development of cancer, it has been suggested that high levels of STB may play a protective role against carcinogenesis [10,11]. 

Previous prospective studies looking at the association between pre-diagnostic levels of STB and site-specific cancer risk have revealed mixed results [12,13,14]. Most studies have specifically explored the association between colorectal (CRC) and lung cancer risk, and bilirubin levels. Recent studies showed that STB levels were inversely associated with colorectal cancer risk [12,13,14]. In addition, three cohort studies also found an inverse association with lung cancer risk [15,16,17]. To our knowledge, no studies investigating the association between STB and overall cancer risk have been performed. A cancer endpoint, which has been studied in relation to STB is total cancer mortality. A large population-based study from Belgium found that baseline levels of bilirubin were inversely associated with overall cancer mortality [18]. Various studies have looked at the association between other circulating antioxidants (i.e., albumin and uric acid) and risk of cancer with mixed results. While albumin levels have mostly been inversely associated with the risk of cancer [19,20,21,22], uric acid has been associated with an increased risk of cancer and increased cancer-related mortality [22,23,24,25]. Therefore, further studies exploring the association between endogenous antioxidants, including bilirubin and cancer risk, are needed.

In the current study, we aimed to analyze pre-diagnostic levels of STB in relation to the development of overall, as well as site-specific, cancer using data from the Swedish Apolipoprotein Mortality Risk (AMORIS) cohort. Additionally, we performed a systematic review and meta-analysis of the existing literature to further evaluate these associations.

## 2. Materials and Methods

### 2.1. AMORIS Cohort

#### 2.1.1. Study Population and Data Collection

Our study population consisted of participants enrolled in the AMORIS cohort, with over 800,000 men and women, mainly from the greater Stockholm area, who underwent health examinations between 1985 and 1996 [26]. They were either healthy individuals referred for clinical laboratory testing as part of a general health check-up, or outpatients referred for laboratory testing [27]. All laboratory analyses were performed at the Central Automation Laboratory (CALAB) in Stockholm. None were inpatients at the time of their blood sample analysis. A more detailed description of the AMORIS cohort can be found elsewhere [26,27,28]. Data on history of any prior cancer diagnosis, clinical outcomes, and sociodemographic information was determined through linkage with the Swedish National Cancer Register, the National Cause of Death Register, the National Patient Register, the National Register of Emigration and the consecutive Swedish Censuses during 1970–1990 by using a unique Swedish 10-digit personal identification number [26,27,28]. As such, the AMORIS cohort provides comprehensive information on cancer diagnosis, socioeconomic factors, co-morbidities and mortality. This study complied with the Declaration of Helsinki, and the ethics review board of the Karolinska Institute approved the study.

Eligible participants in the current study were men and women, aged 20 years or older, who had no record of prior cancers in the National Cancer Register. Subjects with no baseline measurement of serum bilirubin available were excluded from this study. Participants diagnosed with cancer within the first year of study commencement were also excluded, to account for reverse causation, resulting in a final study sample of 137,045 men and women. In addition to overall cancer, we also investigated major cancer types (i.e., those with > 1000 cases during the study follow-up). These included: colorectal (CRC), lung (LC), breast (BC), gynecological (GYN), prostate, bladder, melanoma and hematological cancers.

For the purpose of this study, we used the following information: age at measurement, serum total bilirubin (STB, µmol/L), fasting status, body mass index (BMI), socioeconomic status (SES), education level, Charlson Comorbidity Index (CCI), cancer diagnosis, death and emigration dates. STB was measured within bile pigment using a diazo reagent to produce azobilirubin. 

#### 2.1.2. Data Analysis

Multivariate Cox proportional hazards models were used to calculate hazard ratios (HRs) and 95% confidence intervals (CI) for overall and site-specific cancer risk. Follow-up time was defined from date of bilirubin measurement until date of cancer diagnosis, death from any cause, emigration or end of study (31 December 2012), whichever occurred first.

**Figure 2:**
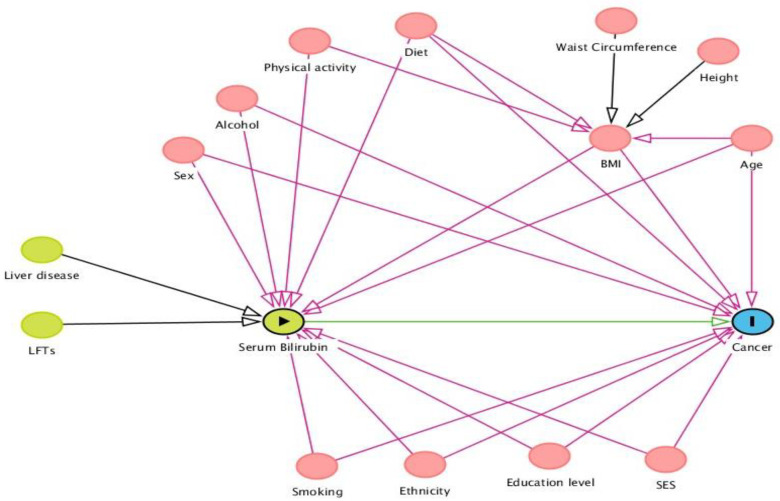
Directed acyclic graph to assess relationships between potential confounders. BMI, body mass index; SES, socioeconomic status; LFTs, liver function tests. Serum bilirubin is the exposure of interest, whilst cancer is the outcome. The other variables are hypothesized confounders of the association between exposure and outcome. The green arrow illustrates the exposure-outcome relationship; pink arrows illustrate the confounder-exposure and confounder-outcome relationships; black arrows illustrate associations between variables with no confounding effect; blue arrows illustrate potential effect modifiers between exposure and outcome.

For the analyses, STB was examined both as a continuous variable and a categorical one (using quartiles). Guided by a directed acyclic graph (DAG) to assess relationships between potential confounders (Figure 2), we developed an adjusted model to examine the relationship between STB and overall and site-specific cancer risk. The following covariates (assessed at baseline) were included as confounders in our final model: age at measurement (continuous), sex (male, female), education level (low, middle, high, missing), CCI (0, 1, 2, 3+) and BMI. Sex was only treated as a confounder and/or effect modifier for those cancers that affect both genders (i.e., prostate). The following confounding factors could not be included in our analysis since the percentages of participants with these measurements were low: physical activity, diet and alcohol consumption. For lung cancer specifically, we performed a sensitivity analysis stratifying by smoking status (i.e., ever smokers and never smokers). In addition, sex-stratified models were conducted to assess effect modification in overall cancer risk. All analyses were conducted using STATA, Version 16 (College Station, Texas, USA; accessed on 1 June 2021).

### 2.2. Systematic Review and Meta-Analysis

#### 2.2.1. Literature Search Strategy

The current study was conducted in accordance with the Preferred Reporting Items for Systematic Reviews and Meta-analyses (PRISMA) guidelines. No protocol has been registered for the study. A literature search of epidemiological studies was conducted on 15 May 2021 using the search engine PubMed, a bibliographic database including over 30 million references to journal articles, with the following search terms: (“Neoplasms”[Mesh] OR “cancer risk”[Title/Abstract]) AND (“Hyperbilirubinemia”[Mesh] OR “bilirubin”[Mesh] OR “bilirubin levels”[Title/Abstract]). The search was restricted to human studies published from January 1955 to May 2021. The inclusion criteria considered studies on adults only. No restrictions were placed on publication type. Non-English publications, duplicate studies, preprints, errata and animal studies were excluded. Moreover, only publications with full text available were included.

Publications were initially screened by title and abstract, with potentially relevant studies undergoing a full-text review. Following the inclusion and exclusion criteria described above, some studies were further excluded in the full-text review. No publications on survival or therapeutics were included.

For our meta-analysis, the following set of inclusion criteria was used to select the final set of studies: the publication pertained to an epidemiological study, which measured STB levels in association with any site-specific cancer risk; the type of cancer was defined as well as the measurement and values of STB; the analytical methods were all described; the study was performed on the whole population (i.e., not stratified by male/female). To include studies of large enough power, only those with at least 20 cancer cases were included. Initially, in the screening phase, titles and abstracts of articles were reviewed to ascertain whether they might potentially fit the inclusion criteria. If, after assessing the abstract, there was any doubt over whether it met the relevant criteria, it was subjected to a subsequent assessment. The list of potential articles was further shortened by performing a detailed evaluation of the methods and results of each of the remaining papers. In addition, we included results from the analysis we performed in the AMORIS cohort. The following details were recorded for each study included in our meta-analysis: author, year of publication, country where the study was undertaken, study type, population, exposure variable, outcome (i.e., cancer type), findings and adjustments.

#### 2.2.2. Meta-Analysis Statistical Techniques

The effect of STB on site-specific cancer risk was evaluated by calculating the random effects summary relative risk to allow for possible heterogeneity between study results. The effect was only analyzed for CRC, breast and lung cancers as our search did not find enough studies for other tumor types that met the inclusion criteria. If a study reported both clinical cut-off and Q4, Q4 was used. Studies using STB continuous values were also included. Potential heterogeneity of the study results was assessed with a forest plot. Potential heterogeneity of the study results was also statistically evaluated using the I2 statistic. All analyses were performed using STATA, version 16 (College Station, TX, USA; accessed on 1 June 2021).

## 3. Result

### 3.1. AMORIS Cohort

A total of 27,695 participants in the study developed cancer during a median follow-up time of 19 years. The baseline participant characteristics by cancer status are shown in Table 1. The results of the multivariate Cox proportional hazards regression (adjusted for age, sex, BMI and education) examining the association between STB and cancer risk, in both the overall population and stratified by sex, are shown in Table 2. No associations were found between STB levels, using a continuous variable and stratified by quartiles, and overall cancer risk for the whole population or when stratifying by sex. Results of further analyses examining STB and risk of site-specific cancer (i.e., CRC, lung, breast, GYN, prostate, bladder, melanoma and hematological) are shown in Table 3. A consistent inverse association was found between STB and risk of lung (HR for Q4 vs. 1st quartile (Q1): 0.50; 95%CI 0.44–0.59) cancer. When stratifying by smoking status, this association persisted in non-smokers (*n* = 21,963, HR for Q4 vs. Q1: 0.45; 95%CI 0.24–0.86), while no significant associations were found in smokers (*n* = 5470, Table 4). Moreover, an inverse association was also found between high STB levels and gynecological cancer (HR for Q4 vs. Q1: 0.86; 95%CI: 0.76–0.99). On the other hand, a positive association was found between high STB levels and both risk of breast cancer (HR for Q4 vs. Q1: 1.13; 95%CI 1.01–1.25) and melanoma (HR for Q4 vs. Q1: 1.24; 95%CI 1.05–1.46). No other significant associations were found between STB and other site-specific cancers.

### 3.2. Meta-Analysis

**Figure 3:**
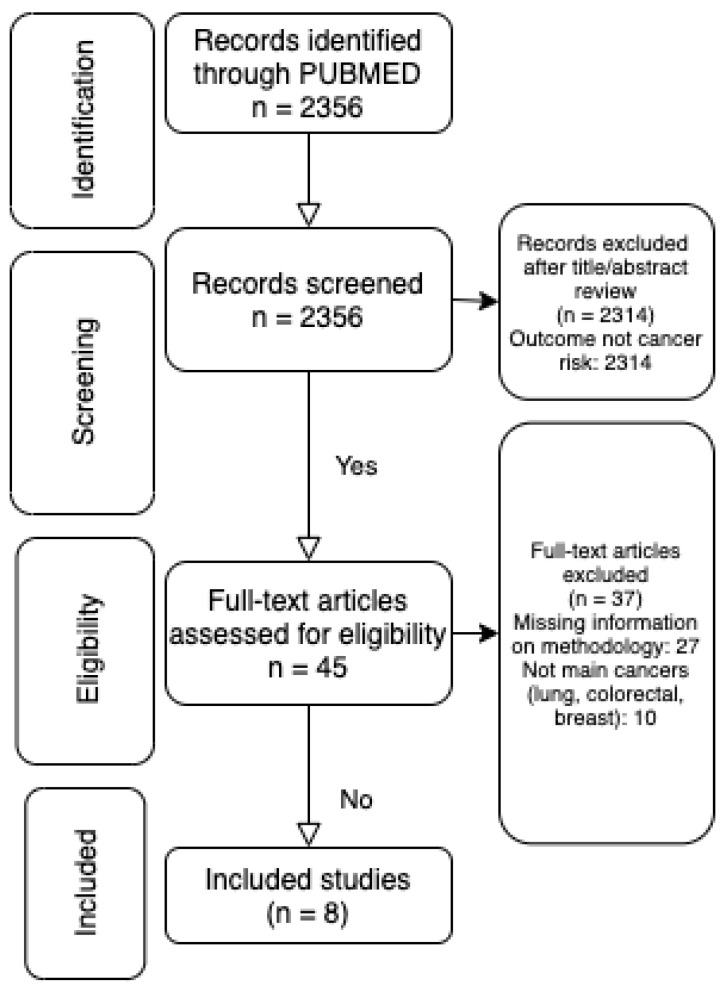
PRISMA diagram representing the review strategy.

The initial search for papers on STB and cancer risk (any cancer) resulted in 45 articles. Eighteen articles were found looking at site-specific cancers (Figure 3). After extracting information from the full-text articles, eight studies looking at CRC, LC and BC risk, that met our inclusion criteria, were included in our meta-analysis. A detailed overview of these studies is given in Table 5. 

**Figure 4:**
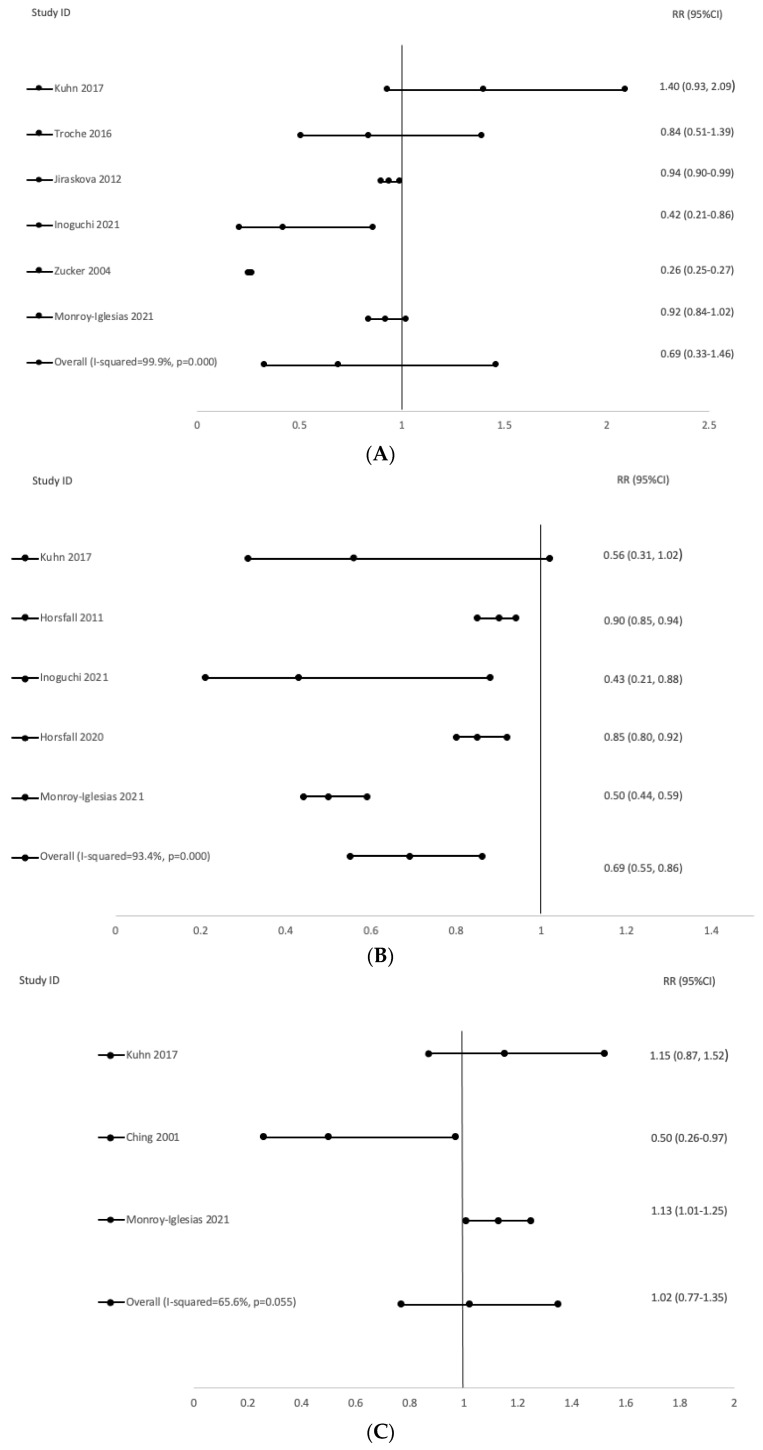
Heme Forest plot for the association between elevated serum total bilirubin (STB) and cancer risk for (**A**) Colorectal cancer, (**B**) Lung cancer, and (**C**) Breast cancer.

The random effects analysis comparing CRC cancer risk and STB quartiles did not show any significant associations, with a pooled effect relative risk (RR) of 0.69 (95%CI: 0.0.33–1.46). The I2 statistic detected substantial heterogeneity between studies (I2 = 99.9%, *p* = 0.000), which can also be observed in the corresponding forest plot (Figure 4A). On the other hand, we observed a significant inverse association between STB and lung cancer risk, where the pooled relative risk was 0.69 (95%CI: 0.55–0.86). The I2 statistic (I2 = 93.4%; *p* = 0.000) also suggested heterogeneity (Figure 4B). Lastly, the pooled relative risk for breast cancer risk did not show a significant association with high STB levels (RR: 1.02; 95%CI: 0.77–1.35) with an I2 statistic of 65.6% (*p* = 0.055; Figure 4C).

## 4. Discussion

In our prospective study, we found no significant association between STB levels and risk of overall cancer. When analyzing major cancer types, our cohort study found a strong inverse association between STB and lung cancer risk that remained for the non-smoking population in a sensitivity analysis. This association was supported by our meta-analysis. An inverse association was also found between STB levels and risk of gynecological cancer. In addition, a positive association was found with the risk of melanoma and breast cancer. However, no association was found between STB and breast cancer risk in the meta-analysis. No significant associations were found for other cancer types.

To our knowledge, this is the first observational study analyzing the association between STB levels and overall cancer risk. It is also by far the largest prospective study of STB and overall cancer risk with a long-term follow-up using high-quality Swedish national registers. Results of prior studies looking at STB and the risk of site-specific cancer types have been inconsistent. A large population-based study by Kuhn et al. found no significant associations between serum bilirubin and risk of breast, prostate, CRC and lung cancer [11]. However, large prospective studies have reported a strong inverse association between STB and lung cancer risk [15,17,29]. This was in line with the present study, where both our cohort study and the meta-analysis found an inverse association between STB and lung cancer risk. Various population-based cohort studies have also linked raised STB with lower rates of overall respiratory diseases, in addition to lung cancer, with the strongest associations in cigarette smokers [11,17,29]. A large cohort study by Wen et al. described that while smoking is a strong risk factor for lung cancer, the smoking-related risk is particularly high among male smokers with low levels of bilirubin (55% increase among those with bilirubin <0.75 mg/dL) [16]. Thus, serum bilirubin may be useful to identify smokers at particularly high risk for lung cancer. However, the current study only found a significant association in non-smokers. This may be explained by the high number of missing data on smoking for cancer cases (>90%). Moreover, most well-established risk factors for cancer (e.g., smoking, obesity, ionizing and ultraviolet radiation) are likely associated with increased reactive oxygen species (ROS). An increase in ROS has been linked to damage in DNA, thus promoting carcinogenesis. Since bilirubin is a strong antioxidant, low STB levels may result in an impaired defense against oxidative stress-induced damage [30]. Moreover, a recent study using machine learning to analyze a non-linear relationship between STB and cancer reported an inverse association with lung cancer risk [30]. In this study, subjects with high STB levels were thought to be those with Gilbert’s syndrome, where the frequency of phenotypic hyperbilirubinemia is estimated to be up to 10% of the population [5]. This association was additionally supported by Horsfall et al., who reported that genetically-raised serum bilirubin may protect people exposed to high levels of smoke oxidants associated with lung cancer [15].

Furthermore, there is increasing evidence for the association between STB and CRC incidence. However, results remain inconclusive. A recent prospective study by Zucker et al., using data from the Third National Health and Nutrition Examination Survey (NHANES), found a positive association between bilirubin levels and risk of CRC cancer in men, and an inverse association in women. In addition, the authors performed a Mendelian randomization analysis of the main UGT1A1 single-nucleotide polymorphism (SNP) associated with Gilbert’s Syndrome. These analyses reported a positive association between high bilirubin and CRC risk in men, while there was no association in women [1]. On the other hand, four epidemiological studies found an inverse association between risk of CRC and STB levels [3,12,14,30]. The results from the current study are in line with those from three other observational studies, which found no significant association between STB and CRC cancer [10,11,31]. The inconsistencies observed in these epidemiological studies are likely to be caused by methodological differences, including study design and sample size. In addition, CRC has been linked to liver failure and, therefore, hyperbilirubinemia due to liver metastases [32]. Further studies looking into the association between STB and CRC risk are required. Moreover, few epidemiological studies have investigated the association between STB and other site-specific cancers. A case-control study looking at serum levels of diet-derived micronutrients and antioxidants, and endogenous antioxidants, in relation to breast cancer risk found a strong inverse association [33]. However, results from the current study are in line with the previously mentioned cohort by Kuhn et al., where no significant associations were found between high STB levels and breast cancer risk [11]. Further studies analyzing possible biological mechanisms behind these associations are warranted. Lastly, a recent study looking at the melanoma cell line found that bilirubin induced apoptotic pathways in cancer cells [34]. This was in line with our current findings.

The strengths of our cohort study include the cohort size with 19 years of follow-up (on average) and reliable outcome data prospectively collected through linkages with national registries. New cases of cancer were identified using the Swedish National Cancer Register with a mandatory reporting of diagnosed cases. Furthermore, STB was measured at the same clinical laboratory for all subjects using a consistently implemented and well-documented methodology. One limitation of our cohort study was that the exposure variables were measured only once (at baseline), so potential changes over time could not be accounted for. Various confounding factors could not be included in our analysis due to the low percentage of participants with measurements (i.e., diet, alcohol consumption, physical activity). In addition to needing more data on smoking status, a more detailed account of smoking characteristics (i.e., packs, years) would have been useful to perform further stratified analyses in the association between STB and lung cancer risk, as the current study only examined never vs. ever smokers (no data on smoking intensity). In addition, measures on UCB were not included in the AMORIS cohort. Finally, given the homogeneous ethnic background of our cohort population (mainly Caucasian; 85% Swedish born), we were limited in our exploration of other ethnic groups. For the meta-analysis, we made all possible efforts to include all relevant publications available to date through PubMed, one of the main online databases. However, one major limitation was the low number of studies fulfilling our inclusion criteria. In addition, there was high heterogeneity between studies, which may have been due to differences in study design. Future studies should further explore the association between STB and risk of cancer, in particular risk of lung cancer, taking a better look into the role smoking status plays in the association.

## 5. Conclusions

Our findings suggest no association between STB levels and risk of overall cancer. However, a consistent inverse association was found between high levels of STB and risk of lung and gynecological cancer. In addition, a positive association was found with risk of melanoma and breast cancer. The results from our meta-analysis supported the association between STB levels and lung cancer, while no association was found with risk of CRC or breast cancer. Additional studies analyzing the association between STB and site-specific cancers are needed—including preclinical studies and those with detailed information on smoking.

## Figures and Tables

**Table 1:** Descriptive statistics of the selected AMORIS cohort, overall and divided by cancer status.

	Non-Cancer (*n* = 109,350)	Cancer (*n* = 27,695)	Total (*n* = 137,045)
Sex			
Male	58,800 (53.8)	15,498 (56)	74,298 (54.2)
Female	50,550 (46.2)	12,197 (44)	62,747 (45.8)
Age (Median, SD)	46.3 (14.9)	53.3 (12)	11.3 (7)
SES			
Low	48,145 (44)	11,450 (41.3)	59,595 (43.5)
Medium	48,643 (44.5)	13,826 (49.9)	62,469 (45.6)
High	12,562 (11.5)	2419 (8.7)	14,981 (10.9)
Bilirubin			
Continuous	11.3 (7.2)	11.4 (5.9)	
Q1	35,214 (32.2)	7982 (28.8)	43,196 (31.5)
Q3	22,645 (20.7)	6089 (22)	28,734 (21)
Q3	25,612 (23.4)	6983 (25.2)	32,595 (23.8)
Q4	25,879 (23.7)	6641 (24)	32,520 (23.7)
Number of comorbidities			
0	101,058 (92.4)	25,533 (92.2)	126,591 (92.4)
1	5582 (5.1)	1562 (5.6)	7144 (5.2)
2	1537 (1.4)	356 (1.3)	1893 (1.4)
≥3	1173 (1.1)	244 (0.9)	1417 (1.0)
Fasting status			
No	34,336 (31.4)	8554 (30.9)	42,890 (31.3)
Yes	52,628 (48.1)	15,394 (55.6)	68,022 (49.6)
NK	22,386 (20.5)	3747 (13.5)	26,133 (19.1)
Education category			
1	30,861 (28.2)	8842 (31.9)	39,704 (29)
2	44,618 (40.8)	10,978 (39.6)	55,596 (40.6)
3	26,762 (24.5)	6346 (22.9)	33,108 (24.2)
NK	7108 (6.5)	1529 (5.5)	8637 (6.3)
BMI (mean, SD)			
	24.6 (5.7)	24.9 (3.9)	24.6 (5.4)
≤24.9	5810 (5.3)	1326 (4.8)	7136 (5.2)
25–29.9	2963 (2.7)	865 (3.1)	3828 (2.8)
≥30	767 (0.7)	212 (0.8)	979 (0.7)
Missing	99,810 (91.3)	25,292 (91.3)	125,102 (91.3)

BMI, body mass index; NK, not known; Q, quartile; SD, standard deviation.

**Table 2:** Hazard ratios (HR) and 95% confidence intervals (CI) for continuous and quartiles of serum total bilirubin (STB), by overall cancer risk and stratified by sex.

	Overall N = 27,695		Men N = 15,498		Women N = 12,197	
	HR	95%CI	HR	95%CI	HR	95%CI
Continuous	0.99	0.99–1.00	0.97	0.94–1.01	0.99	0.98–1.00
Q1 ≤8	1.00	Ref	1.00	Ref	1.00	Ref
Q2 9–10	0.97	0.94–1.00	1.00	0.82–1.22	0.99	0.94–1.04
Q3 11–13	0.96	0.92–0.99	0.85	0.70–1.04	1.00	0.95–1.05
Q4 ≥14	0.96	0.93–1.00	1.07	0.90–1.29	0.98	0.93–1.04

Q, quartile; HR, hazard ratio; CI, confidence interval.

**Table 3:** Hazard ratios (HR) and 95% confidence intervals (CI) for continuous and quartiles of serum total bilirubin (STB), by overall cancer risk and stratified by sex.

	Continuous	Q1 ≤8	Q2 9–10	Q3 11–13	Q4 ≥13
	HR (95%CI)	HR (95%CI)	HR (95%CI)	HR (95%CI)	HR (95%CI)
Any Cancer	0.99 (0.99–1.00)	1.00 (Ref)	0.97 (0.94–1.00)	0.96 (0.92–0.99)	0.96 (0.93–1.00)
Colorectal	0.99 (0.98–1.00)	1.00 (Ref)	0.94 (0.85–1.03)	0.91 (0.82–1.00)	0.92 (0.84–1.02)
Lung	0.94 (0.93–0.95)	1.00 (Ref)	0.74 (0.64–0.84)	0.66 (0.58–0.75)	0.50 (0.44–0.59)
Breast	1.00 (0.99–1.01)	1.00 (Ref)	1.09 (1.00–1.20)	1.06 (0.97–1.17)	1.13 (1.01–1.25)
Gynaecological	0.99 (0.98–1.00)	1.00 (Ref)	1.02 (0.92–1.14)	0.97 (0.86–1.09)	0.86 (0.76–0.99)
Prostate	1.00 (0.99–1.00)	1.00 (Ref)	0.94 (0.86–1.03)	0.97 (0.89–1.05)	1.00 (0.93–1.09)
Bladder	0.99 (0.97–1.99)	1.00 (Ref)	0.89 (0.75–1.07)	0.88 (0.75–1.05)	0.91 (0.77–1.08)
Melanoma	1.01 (1.00–1.01)	1.00 (Ref)	1.15 (0.97–1.35)	1.08 (0.92–1.28)	1.25 (1.06–1.47)
Haematological	1.00 (0.99–1.01)	1.00(Ref)	1.06 (0.92–1.23)	0.94 (0.81–1.08)	1.09 (0.95–1.26)

Q, quartile; HR, hazard ratio; CI, confidence interval.

**Table 4:** Hazard ratios (HR) and 95% confidence intervals (CI) for continuous and quartiles of serum total bilirubin (STB) and lung cancer risk, stratified by smoking status.

	Overall N = 1875		Smokers N = 82		Non-Smokers N = 102	
Bilirubin levels						
Mean (SD)	10.3 (4.6)		10.1 (8.4)		10.3 (3.6)	
	HR	95%CI	HR (N)	95%CI	HR (N)	95%CI
Continuous	0.94	0.93–0.95	1.00	0.96–1.04	0.92	0.87–0.97
Q1 ≤8	1.00	Ref	1.00 (29)	Ref	1.00 (40)	Ref
Q2 9–10	0.73	0.64–0.84	0.59 25	0.31–1.10	0.93 (14)	0.54–1.61
Q3 11–13	0.65	0.58–0.75	0.53 33	0.27–1.03	1.02 (15)	0.61–1.70
Q4 ≥14	0.50	0.44–0.59	0.84 15	0.44–1.60	0.45 (13)	0.24–0.86
p-trend	0.000		0.241		0.040	

SD, standard deviation; HR, hazard ratio; CI, confidence interval; Q, quartile; N, number of patients in category.

**Table 5:** Overview of observational studies included in our meta-analysis.

Author, Year	Country	Study Type	Population	Follow-Up	Exposure	Outcome	Findings	Confounders/Adjustments	Observations
Kuhn, 2017	EPIC	Cohort	627 BC, 554 PC, 195 LC, 256 CRC cases	14.8	Serum bilirubin	BC, PC, CRC, LC risk	No significant associations	Age, smoking, alcohol, aspirin use, PA, WC, BMI, height, education level. BC +hormonal factors CRC + diet	Also looking at albumin and uric acid
Inoguchi, 2021	Japan	Cohort	403 LC; 315 CRC	4.7	Serum bilirubin	LC, CRC, BC, PC, cervical risk	Decreased LC risk in men (HR 0.47; 95%CI 0.27–0.82); no significant association for women. Decreased CRC risk (HR 0.42; 95%CI 0.21–0.86).		
Troche, 2016	USA (PLCO, NCAS)	Case-Control (2)	PLCO- 252 cases, 250 controls; NCAS- 120 cases, 77 controls	15	Serum bilirubin stratified by alcohol consumption	CRC risk	No significant associations in either study	Age, sex and smoking	
Jirásková, 2011	Czech Republic	Case-Control	777 cases and 986 controls	NK	Serum bilirubin	CRC risk	Decreased CRC risk (OR 0.94; 95%CI 0.90–0.99) Decreased CRC risk men (0.93; 95%CI 0.87–0.99) Decreased CRC risk women (0.92; 95%CI 0.85–1.00, *p* = 0.05)	Sex and age	Per mmol increase of serum bilirubin
Zucker, 2004	USA	Cohort	83 cases	NK	Serum bilirubin	CRC risk	Decreased CRC risk (OR 0.257; 95%CI 0.254–0.260)		Per 1 mg/dL increase in bilirubin levels.
Horsfall, 2011	USA	Cohort	1341 cases	8.3	Serum bilirubin	LC risk	Decreased LC risk in men (IRR 0.92; 95%CI 0.89–0.95) Decreased LC risk in women (IRR 0.89; 95%CI 0.86–0.93)	Age, BMI, systolic blood pressure, smoking status, alcohol intake and SES	Per 0.1 mg/dL increase in bilirubin
Horsfall, 2020	UK	Cohort	2002 cases	NK	Serum bilirubin	LC risk	Decreased LC risk (IRR 0.85; 95%CI 0.80–0.92)	Age, gender, calendar year, ethnicity, height, weight, recruitment centre and smoking status	Per 5 nmol/L increase in bilirubin levels.
Ching, 2001	Australia	Case-Control	153 cases, 151 controls	NK	Serum bilirubin	BC risk	Decreased BC risk (OR 0.50; 95%CI 0.26–0.97)	Age at menarche, parity, dietary fat and alcohol intake	

BC, breast cancer; PC, prostate cancer; LC, lung cancer; CRC, colorectal cancer; PA, physical activity; WC, waist circumference; BMI, body mass index; HR, hazard ratio; CI, confidence interval; OR, odds ratio; NK, not known; IRR, incidence rate ratio.

## Data Availability

The data presented in this study are available on request from the corresponding author. The data are not publicly available due to ethical reasons.
